# An economic analysis of email-based telemedicine: A cost minimisation study of two service models

**DOI:** 10.1186/1472-6963-8-107

**Published:** 2008-05-22

**Authors:** Liam Caffery, Anthony C Smith, Paul A Scuffham

**Affiliations:** 1 Centre for Online Health, University of Queensland, Australia, Level 3 Foundation Building. Royal Children's Hospital, Herston Road, Herston, Queensland 4029, Australia; 2 School of Medicine, Griffith University, Logan Campus L03 2.43. Griffith University, Queensland, Australia

## Abstract

**Background:**

Email-based telemedicine has been reported to be an efficient method of delivering online health services to patients at a distance and is often described as a low-cost form of telemedicine. The service may be low-cost if the healthcare organisation utilise their existing email infrastructure to provide their telemedicine service. Many healthcare organisations use commercial-off-the-shelf (COTS) email applications. COTS email applications are designed for peer-to-peer communication; hence, in situations where multiple clinicians need to be involved, COTS applications may be deficient in delivering telemedicine. Larger services often rely on different staff disciplines to run their service and telemedicine tools for supervisors, clinicians and administrative staff are not available in COTS applications. Hence, some organisations may choose to develop a purpose-written email application to support telemedicine. We have conducted a cost-minimisation analysis of two different service models for establishing and operating an email service. The first service model used a COTS email application and the second used a purpose-written telemedicine application.

**Methods:**

The actual costs used in the analysis were from two organisations that originally ran their counselling service with a COTS email application and later implemented a purpose-written application. The purpose-written application automated a number of the tasks associated with running an email-based service. We calculated a threshold at which the higher initial costs for software development were offset by efficiency gains from automation. We also performed a sensitivity analysis to determine the effect of individual costs on the threshold.

**Results:**

The cost of providing an email service at 1000 consultations per annum was $19,930 using a COTS email application and $31,925 using a purpose-written application. At 10,000 consultations per annum the cost of providing the service using COTS email software was $293,341 compared to $272,749 for the purpose-written application. The threshold was calculated at a workload of 5216 consultations per annum. When more than 5216 email consultations per annum are undertaken, the purpose-written application was cheaper than the COTS service model. The sensitivity analysis showed the threshold was most sensitive to changes in administrative staff salaries.

**Conclusion:**

In the context of telemedicine, we have compared two different service models for email-based communication – purpose-written and COTS applications. Under the circumstances described in the paper, when workload exceeded 5216 email consultations per annum, there were savings made when a purpose-written email application was used. This analysis provides a useful economic model for organisations contemplating the use of an email-based telemedicine system.

## Background

Email-based telemedicine is often described as a low-cost form of telemedicine [1-3]. This may be true if the telemedicine service uses existing email infrastructure; however, a number of authors have identified that commercial-off-the-shelf (COTS) email applications are deficient at running telemedicine services and an email-based telemedicine service is more efficient using a purpose-written telemedicine application [4,5]. We developed a purpose-written application for email-based telemedicine services. Our purpose-written application is a webmail product designed to support the workflow of supervisors, clinicians and administrative staff of the telemedicine provider. It was developed after requirements analysis was performed for the subject organisations. Telemedicine functionalities of our webmail application are described below:

▪All incoming emails are automatically scanned and flagged if they contained pre-defined keywords.

▪Email cases can be triaged and assigned to a particular clinician.

▪Auditing tools ensure cases are answered in a timely fashion.

▪Full patient history can be viewed online.

▪Complex cases can be flagged by the clinician, ensuring a supervisor endorses the response, before it is sent to the client.

Our application also automates a number of tasks associated with running a telemedicine service – for example, the automatic allocation of cases to a clinician with an existing relationship with a client and the automatic provision of workload statistics.

We implemented our purpose-written application for two counselling organisations. The first organisation is a national telephone and email counselling service aimed specifically for children aged five to eighteen years. The service is free and staffed by over 100 professional counsellors – 90% of whom have tertiary qualifications in psychology, social work and related disciplines [6]. Most client emails are regarding family and peer relationships. Email contact relating to mental health, suicide, eating behaviours and self-image are to two to three times higher than for telephone counselling [6] – indicating clients enjoy the anonymity of email.

The second subject organisation is a registered charity that provides support services to family carers. Carers are mostly family members who provide voluntary care for a person who is aged, with a disability or with a chronic or mental illness. Caring places enormous demands on these carers and the negative consequences associated with caring are well documented [7]. It has also been well documented that carers who seek intervention have less depression, less stress and significant improvement in measures of vitality, social functioning and mental health [7-10]. For these reasons, counselling is an important support service offered by the charity.

Following the implementation of our purpose-written telemedicine software for these two email counselling organisations in Australia [11], the present study compares the economics of both of these services. We have analysed the costs of establishing and operating a telemedicine service using COTS email software and compared these with the costs of establishing and operating the same telemedicine service with our purpose-written email-based telemedicine application.

The aim of this study was to identify costs associated with providing an email-based telemedicine services using two alternative service models and determine the conditions required for each service to become economically viable.

## Methods

We compared the actual costs of operating an email-based telemedicine service for the two service models. Both organisations involved in our study were operating an email-based service using COTS software prior to implementing our purpose-written application. The fixed and variable costs for both service delivery alternatives were calculated from data collected from both organisations. By evaluating two organisations we were able to more accurately determine variable costs at a range of different workloads.

### Data Collection

Workload activity data was collected from both counselling organisations (Table 1). Our subject organisation recorded information about all email interactions in a database, including the amount of time counsellors spent on each teleconsultation and the service delivery model. Data extracted from this database was used for counsellor cost calculations.

**Table 1:** Telemedicine activity for the two counselling organisations.

	***Organisation A***	***Organisation B***
Study Period	May 2004 – Aug 2006	Jun 2005 – Jun 2006
Total duration (months)	28	13
Total number of email consultations	21,207	369
Average consultations per month	784	30
Consultations per month – range	611–1097	6–53

Administrative and supervisory staff recorded the time they spent on the email service pre- and post- installation of the purpose-written application. Results from this study form the basis of staff cost calculations for these disciplines [11].

Capital costs and service fees were actual costs we charged the counselling organisation for developing and hosting the purpose-written email application.

### Assumptions

The present cost analysis was based on the assumption that patient outcomes were the same for both service alternatives. Costs were calculated from the perspective of the healthcare organisation in Australian dollars (A$1 =~US$0.86 or ~0.63 Euro) and included 10% Goods and Services Tax. Staff pay rates in both counselling organisations were calculated according to the state health department's professional and administrative officer's scale. We added an additional 20% to the salary expenses, to account for the employee's non-salary costs or "on-costs" – for example, superannuation and work cover premiums.

It was unnecessary to employ all disciplines of staff for all workload volumes – for example, if only one counsellor was employed it would be unwarranted to also employ a supervisor; similarly a small volume service may not require administrative support. Staff involved in the delivery of email counselling service suggested the appropriate workload volume to introduce administrative support and supervisory support was 3000 and 6000 email consultations per annum respectively and only one person from each discipline was necessary until the workload exceed 10,000 email consultations per annum.

The annual service fee for the purpose-written email application included minor enhancements and maintenance programming. For these reasons, it was assumed the software would remain operational for seven years.

### Staff Costs

A counsellor's salary was based on the Professional Officer Level 2.6 rate of $67,670 per annum per full time equivalent, including on-costs. Their duties included reading and responding to client emails.

A supervisor's duties include triage of all incoming emails to assign a priority, allocation of cases to staff and proofing outgoing responses from counsellors. A supervisor salary was based on the Professional Officer Level 3.4 rate of $77,842 per annum per full time equivalent, including 20% on-costs.

Administrative staff costs were based on the Administrative Officer Level 2 Increment 8 pay scale or $49,177 per annum per full-time equivalent, including on-costs. Their duties include retrieving patient history when an email was from existing client, auditing client emails to ensure all incoming emails had been answered and the manual provision of workload data for management reports.

### Fixed Costs

Fixed costs included capital costs to develop and implement the purpose-written email service. The initial capital outlay was converted into an annual equivalent cost through a process of annuitizing these costs over the life expectancy of the application [12]. A life expectancy of seven years and a depreciation rate of 14% was used in the calculations. It was assumed there was no resale value at the end of the lifecycle. In addition, an annual fixed cost (service fee) was also incurred by each counselling organisation when using the purpose-written application. This fee covered the hosting and associated technical support required by the purpose-written application.

### Threshold analysis

The threshold was calculated at which the cost of using the COTS email application was the same as using the purpose written application.

### Sensitivity analysis

A one-way sensitivity analysis was undertaken by varying the key input values by 50% and assessing the change on the threshold where both service delivery options are equal. Key inputs included the cost of developing the purpose-written software, the cost of hosting the software (service fee), and staff salaries.

## Results

### Fixed Costs

The capital cost charged to both counselling organisations to implement our purpose-written application was $30,000, which equated to $6995 after annuitizing. In addition, both counselling organisations incurred an annual fee of $5000 to host the telemedicine application. There were no fixed costs associated with running the service using the COTS software as the original service used the counselling organisation's existing email infrastructure. Fixed costs are summarised in Table 2.

**Table 2:** A summary of the fixed and variable costs associated with running both email counselling services.

	***COTS ($)***	***Purpose-written ($)***
***Fixed costs***		
Annual equivalent cost of capital cost (annual)	0	6995
Service fee (annual)	0	5000
		
***Variable costs***		
Supervisory staff (workload 6000+) (annual)	77,841	61,454
Administrative staff (workload 3000+) (per email)	1.62	0
Counselling staff (per email)	19.93	19.93

### Variable Costs

#### Staff Costs

The main costs associated with providing an email service were for clinical, supervisory and administrative staff salaries.

#### Counsellors

Each counsellor cost the organisation $67,670 per annum. Clinical hours for a counsellor were calculated to be 1357 hours per annum after leave and time for non-clinical duties were subtracted from salaried hours. From the data extract we calculated that a counsellor conducted on average 2.5 email consultations per hour regardless of the service model. This equated to 3395 email consultation per year per counsellor; therefore, the average counselling cost per email is $19.93.

#### Supervisor

When the service was using a COTS application, a supervisor cost the organisation $77,842 per annum, after implementation of the dedicated system, supervisors estimated they saved eight hours per week [11]. Therefore, the supervisor cost using our purpose-written system was $61,454 per annum. Cost for the supervisor was applicable for workloads of 6000 to 10,000 email consultations per annum.

#### Administrative

When the volume of emails was 10,000 consultations per year the administrative officer reported spending 12.5 hours per week on email duties. This equated to $1.62 per email. This cost was applied for workload volumes of 3000 to 10,000 emails per annum. The need for administrative support was eliminated after the introduction of the purpose-written application because of the automation of their duties.

Variable costs are summarised in Table 2.

Total fixed and variable costs are shown in Table 3.

**Table 3:** Total costs associated with operating each email counselling service at varying workloads.

***Email consultations per annum***	***COTS ($)***	***Purpose-written ($)***
1000	19,930	31,925
2000	39,860	51,855
3000	64,650	71,785
4000	86,200	91,715
5000	107,750	111,645
6000	207,141	193,029
7000	228,691	212,959
8000	250,241	232,889
9000	271,791	252,819
10,000	293,341	272,749

#### Threshold

**Figure 1:**
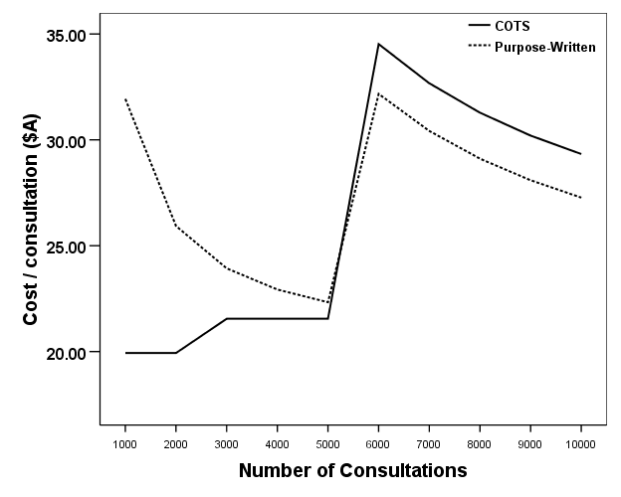


The average cost per email consultation is represented graphically in Figure 1. We calculated the point at which the two lines intersect, by using linear equations, to be between 5000 and 6000 emails consultation per year. Under these conditions, the threshold was reached at a workload of 5216 email consultations per year; when more than 5216 email consultations are undertaken, purpose-written software was cheaper than the COTS application.

#### Sensitivity Analysis

The threshold was re-calculated for a 50% change in each of the key input costs. The key input costs were increased by 25% and decreased by 25% resulting in an overall variation of 50%. The change in threshold was expressed as a ratio to the original base threshold (Table 4). A change of 50% to administrative staff costs resulted in the greatest change to results with a 4.5% change to the original base threshold. All other factors had a lesser effect on results. The factors assessed in this sensitivity analysis had relatively little effect compared with the annual number of emails sent.

**Table 4:** Sensitivity analysis: adjusted threshold calculated when individual cost elements are adjusted by 50%

	*Base Threshold*	*Adjusted Threshold +25%*	*Adjusted Threshold -25%*	*Change*	*Ratio (%)*	*Rank*
Capital cost	5216	5313	5119	194	3.7	2
Service fee	5216	5286	5147	139	2.7	3
Supervisory staff salary	5216	5176	5280	-104	-2.0	4
Administrative staff salary	5216	5102	5336	-234	-4.5	1
Counselling staff salary	5216	5216	5216	0	-	5

## Discussion

Studies of email-based telemedicine have shown that some roles – namely administrative and supervisory – are more automation friendly than clinical roles [11]. Purpose-written email systems – through automation – can reduce or eliminate these roles. This study has determined that when more than ~5200 email consultations are undertaken annually, purpose-written software for use in counselling settings reduces costs through freeing up staff time compared with using COTS. The potential savings increased as workload increased above the threshold value. The threshold was most sensitive to changes in key-input costs (administrative staff costs, capital costs and service fees) affecting only one of the service models. Costs, which affected both service models, resulted in lesser change – for example, counselling staff salary had no effect on the threshold. An adjustment of 50% in costs resulted in maximum change of 4.5% to the threshold hence, effect was considered small. However, the annual volume of emails has a greater impact on results than any of the input factors.

Purpose-written telemedicine applications will often provide benefits over COTS which may be difficult to quantify. These benefits include: ease of implementation – especially in multi-clinician healthcare organisations, auditing tools that ensure no consultation is overlooked and faster turn-around-time to client emails. Healthcare organisations could justify purpose-written telemedicine applications, not only on cost but on these benefits.

Most telemedicine services require start-up costs associated with infrastructure development – for example, purchase of equipment and telecommunications [13-15]. There are similar start-up costs if the healthcare organisation chooses to develop their own purpose-written email application. Interestingly, this study found that the software cost had little effect on the email volume threshold where purpose-written software became cost-saving. Changes in the capital cost (3.7%) to develop the purpose-written software and similarly, the cost of hosting the software (2.7%) were both small. Therefore, developing purpose-written software is a relatively small investment.

This study has compared two options for establishing and running an email service but a healthcare organisation may have a further service delivery option – namely, non-commercial off-the-shelf email applications that have been developed for telemedicine services. Some software authors have made their email-based telemedicine software available free of charge [16-18] through open-source software licensing arrangements. These open-source applications were originally developed for a specific service and therefore may or may not be suitable for another organisation's email service. They would need to be assessed on a case-by-case basis prior to implementation. An organisation would need to determine if one of the open-source applications could be adapted for their own service and whether it provided workflow advantage over COTS applications. They would also need to evaluate the costs of providing IT infrastructure to support it. If an open-source application was found to be suitable, the healthcare organisation could avoid the development cost of a purpose-written application and hence, reduce the threshold number of consultations where a telemedicine specific application is more economical than a COTS application.

Like any economic evaluation, one must clearly state the assumptions which form the basis of the analysis. Our assumptions are based on two subject organisations which provide online counseling services. Caution must be applied in generalising these results to email-based services which support other medical specialties.

## Conclusion

The results show email-based services whose work volume is greater then 5216 emails consultations per year would realise savings by developing their own purpose-written email-based telemedicine application. As workload increases above 5216 consultations, the potential savings would become greater. Alternatively, if the workload is less than 5216 consultations it would be cheaper to implement the service using the organisations existing email system. This analysis provides a useful economic model which demonstrates the relationship between workload and potential savings. The threshold is most sensitive to changes in administrative staff salary. Change to counselling staff salary has no effect on threshold. Developing purpose-written software was found to be a relatively small investment. Before implementing an email-based telemedicine system – health-care providers should consider important factors, which may influence the overall cost to their organisation.

## Competing interests

The authors declare that they have no competing interests.

## Authors' contributions

LC and ACS produced the first draft of the manuscript. ACS and PAS provided valuable guidance with the interpretation of the economic evaluation. All authors contributed to the review and editing of the manuscript. All authors have read and approved the final version of the manuscript.

## Pre-publication history

The pre-publication history for this paper can be accessed here:


